# Current News

**Published:** 1903-11

**Authors:** 


### Current News.

### INSTITUTE OF DENTAL PEDAGOGICS.

Following is the programme of the meeting to be held in Buffalo, December 28, 29, and 30, 1903, at the Iroquois Hotel. All interested in education and the elevation of the standards of the dental colleges and students are very earnestly requested to attend this meeting.

President's Address. "Some Faults of the Prevailing Dental Training." By Dr. J. D. Patterson, Kansas City. Discussion to be opened by Dr. John I. Hart, New York; Dr. B. Holly Smith, Baltimore; Dr. H. P. Carlton, San Francisco; Dr. Geo. E. Hunt, Indianapolis.

Prosthesis. Two papers. (*a*) "Methods of teaching the Artistic Elements of Prosthetic Dentistry." By Dr. A. O. Hunt, Omaha, Neb. (*b*) "Methods of teaching the Anatomical Arrangement of Teeth." By Dr. B. J. Cigrand, Chicago. Discussion to be opened by Dr. N. S. Hoff, Ann Arbor; Dr. G. H Wilson, Cleveland; Dr R. R. Freeman, Nashville; Dr. F. H. Berry, Milwaukee.

"An Ideal in Pathology." Paper by Dr. D. R. Stubblefield, Nashville. Discussion to be opened by Dr. H. A. Smith, Cincinnati; Dr. T. B. Hartzell, Minneapolis; Dr. A. H. Peck, Chicago; Dr O. L. Hertig, Pittsburg.

"Orthodontia Technology." Two papers. By Dr. S. H. Guilford, Philadelphia; Dr. C. S. Case, Chicago. Discussion will be opened by Dr. W. E. Grant, Louisville; Dr. A. E. Webster, Toronto; Dr. H. B. Pullen, Buffalo; Dr. H. T. Smith, Cincinnati.

"The Value of Instruction in Dental History and Literature." Paper by Dr. J. Taft. Discussion to be opened by Dr. H. L. Ambler, Cleveland; Dr. Charles McManus, Hartford; Dr. J. H. Kennerly, St. Louis; Dr. B. J. Cigrand, Chicago.

"Porcelain Technology." Paper by Dr. H. J. Goslee. Discussion to be opened by Dr. J. Q. Byram, Indianapolis; Dr. Ambler Tees, Philadelphia; Dr. L. E. Custer, Dayton; Dr. H. L. Banshaf, Milwaukee; Dr. J. F. Ross, Toronto.

"The Dental Curriculum." Paper by Dr. Geo. E. Hunt, Indianapolis. Discussion to be opened by Dr. G. V. Black; Dr. J. B. Willmott.

"How shall Quizzes be conducted?" Symposium by Dr. F. D. Weisse; Dr. R. H. Nones; Dr. L. P. Bethel.

Exhibition of recent teaching appliances. Dr. W. G. Foster, Baltimore; Dr. L. S. Tenny, Chicago.

W. H. Whitslar,
*Chairman Executive Committee.*

---

### AMERICAN SOCIETY OF ORTHODONTISTS: A CORRECTION.

The meeting of the American Society of Orthodontists will begin on Thursday, December 31, 1903, instead of on Wednesday, December 30, as previously published.

The meeting will be held at the Iroquois Hotel, Buffalo, N. Y., and a most interesting programme has been prepared.

Anna Hopkins,
*Secretary.*

---

### THE FRATERNAL DENTAL SOCIETY OF ST. LOUIS, MO.

Whereas, The Fourth International Dental Congress, which is to meet in St. Louis, August 29 to September 3, 1904, under the auspices of the Louisiana Purchase Exposition, is to be the greatest event in the history of dentistry; and

Whereas, As the Fraternal Dental Society of St. Louis is progressive and stands for the best in dentistry and its interests; therefore be it

*Resolved,* That the Fraternal Dental Society of St. Louis heartily endorses the Fourth International Dental Congress, and tenders it their aid and support as a society and as individuals.

W. J. Whipple, *President pro tem.*

E. E. Haverstick, *Secretary.*

